# Evaluation of Role of Visual Inspection Using Acetic Acid (VIA) and Exfoliative Cytology in Screening and Early Detection of Oral Premalignant Lesions and Oral Cancer

**DOI:** 10.31557/APJCP.2021.22.7.2273

**Published:** 2021-07

**Authors:** Vasudha Singh, Kachnar Varma, Mudita Bhargava, Vatsala Misra, Mangal Singh, Richa Singh

**Affiliations:** 1 *Department of Pathology, M.L.N. Medical College, Allahabad, India. *; 2 *Departement of E.N.T., M.L.N. Medical College, Allahabad, India. *; 3 *Department of Community Medicine, M.L.N. Medical College, Allahabad, India. *

**Keywords:** Acetic acid, exfoliative cytology, early detection, OPMD, OSCC

## Abstract

**Objective:**

Oral cancer accounts for 50–70% of total cancer mortality. VIA screening has drastically changed the morbidity and mortality related to cervical cancer. In present study, we assessed the role of 5% Acetic Acid as a vital stain in oral mucosa of chronic tobacco chewers, to know if it can help in early diagnosis and improved prognosis of oral malignancies and to assess the sensitivity and specificity of VIA and exfoliative cytology for screening of Oral premalignant and malignant lesions in high risk population with limited health care infrastructure.

**Methods:**

This was an outpatient department based prospective study done in a tertiary hospital over a period of two years. 150 cases with a history of chronic tobacco use were evaluated. Visual inspection (VI) followed by VIA using 5% Acetic acid was done. Oral scrapes were taken for cytological examination followed by biopsy for histopathological evaluation, wherever possible.

**Results:**

Sensitivity, specificity, PPV, NPV and accuracy of VIA and Exfoliative cytology was 71%, 65%, 83%, 48%, 69% and 98%, 65%, 89%, 92%, 88% respectively keeping histology as gold standard.

**Conclusion:**

Acetic acid along with exfoliative cytology can be used as simple, cost effective and convenient methods for mass screening of OPMDs and OSCC in high risk population where biopsy is not possible.

## Introduction

Oral cancer is one of the most concerning global health problems. Although representing 2 to 4% of the malignancies in the West, oral cancer accounts for almost 40% of all cancers in the Indian subcontinent. It has high incidence and mortality rates and accounts for 40% of all cancers in India, with a higher incidence in Uttar Pradesh, Madhya Pradesh, Gujarat, Bihar and Maharashtra, attributable to high tobacco consumption in these parts of the country Mehrotra et al., (2003). According to a previous study done at our institute from 1990-2007, the prevalence of oral potentially malignant disorders (OPMD) and oral squamous cell cancer (OSCC) in Allahabad region was 30% and 38% respectively. On an average, 63 new cases of oral cancer per annum were detected during this period (Mehrotra et al., 2008). Thus, due to its high incidence, identifiable precancerous lesions and better outcome upon early recognition and treatment, it is very important to find out methods for mass screening of high risk population. 

Unaided visual inspection (VI) is the most widely practiced method at present for screening in oral cancer. However, individuals with chronic tobacco use have an apparently normal oral mucosa or only subtle changes that may be missed on VI. These patients refuse for invasive procedures such as biopsy for histopathological examination. Such cases eventually present with a higher stage of disease at the time of diagnosis; significantly increasing the morbidity and mortality. Noninvasive modalities like acetic acid application and exfoliative cytology in such cases can be used as a screening and diagnostic tool.

Acetic acid is widely being used as a vital stain for screening of cervical cancer (Sankaranarayanan et al., 2003). It can similarly be used as a simple and cost effective method for mass screening of oral cancer and pre-cancerous lesions. 5% acetic acid has been used as a clinical marker for the detection of oral cancer previously (Bhalang et al., 2008; Vinuth et al., 2015). Our study assessed the efficacy of VIA for early diagnosis of OPMDs and OSCC using exfoliative cytology as an adjunctive technique.

## Material and Methods

This was a prospective study which included 150 patients with history of chronic tobacco consumption for more than five years along with 30 patients as controls without any history of Tobacco use.

**Figure 1:**
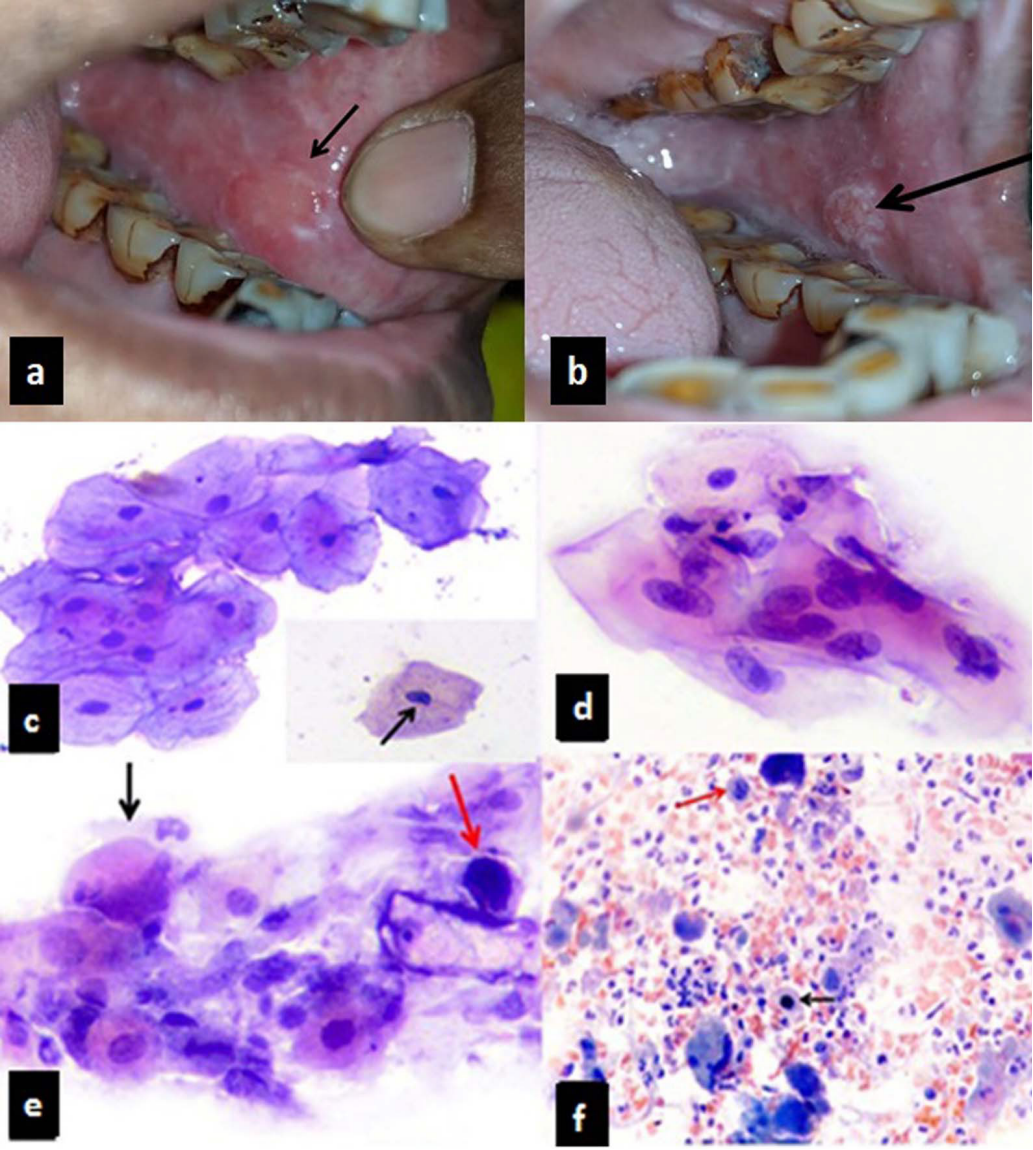
Clinical Image Showing Results of VI, VIA and Cytological Features in Dysplasias and OSCC. a, VI showing erythematous patch on left buccal mucosa; b, Same lesion showing diffuse blanching on VIA; c, ASCUS. Inset- Notching of nuclear membrane (arrow) (PAP, x400); d, LSIL; e, HSIL- Dyskeratosis (black arrow) and atypical mitosis (red arrow) (PAP, X400); f, OSCC-Atypical mitosis (black arrow), multinucleated tumour cell (red arrow) (PAP, x400)

Detailed socioeconomic and demographic data was collected. Visual inspection under adequate illumination was done. This was followed by VIA in which patients were made to rinse the oral cavity with freshly prepared 5% acetic acid for two minutes and presence or absence of blanching of mucosa was noted as shown in Figure 1a-b. The studies on VIA have reported that low specificity can be a limitation of VIA which can lead to referral and treatment of false positive lesions. This was seen in our study in cases where acetic acid concentration was high or increased duration of application was done.

Oral scrapes were then taken for exfoliative cytology using toothbrush technique applying moderate pressure (Mehrotra et al., 2008). The brush while scraping was moved in same direction each time and lesion was scraped till pin point bleeding occurred so as to obtain a transepithelial cellular sample. In VIA positive cases, scrape was done from the blanched area and in patients where there was no blanching, scrape was taken from at least four different sites. The slides with at least 30 well-preserved cells (including intermediate and basal-parabasal cells), not obscured by blood or exudates or necrotic debris were considered adequate for examination (Sankaranarayanan et al., 2003; Mehrotra et al., 2008; Bhalang et al., 2008; Babshet et al., 2011; Vinuth et al., 2015).

All specimens were examined independently by two experienced pathologists in a double-blind manner and findings were noted. Based on the modified Bethesda Classification (Alsaraff et al., 2018) lesions were categorised as Normal, Atypical squamous cells of unknown significance (ASCUS), Squamous Intraepithelial lesion low grade (LSIL), Squamous Intraepithelial lesion high grade (HSIL) and OSCC (Figure 1c-f).

Biopsy from every case could not be obtained because patients with no visible lesion and asymptomatic lesions did not give consent. Out of total 150 cases, biopsy could be obtained from 58 cases only and histopathological examination was done. Biospy was taken from the same site where the lesion was scraped for cytological examination. Most common sites were buccal mucosa, labial mucosa, palate, lateral border and dorsum of tongue. Grading of Dysplasia and Squamous Cell Carcinoma on histopathology was done according to WHO criteria (El-Naggar et al., 2017).

Statistical analysis was done using graph pad, Medcal software and Microsoft office. p Value, sensitivity, specificity, positive predictive value (PPV), negative predictive value (NPV) and diagnostic accuracy of VIA and exfoliative cytology was calculated.

## Results

Of the total cases evaluated, 58% cases were in fourth and fifth decade of life with mean age of 44.6 ± 11.9 years. A predominance of male population was seen with a Male: Female ratio of 6:1. Most of the individuals were from rural background (72%) with history of smokeless tobacco consumption in various forms including gutkha, betel nut, paan. Duration of tobacco consumption was more than 10 years in majority of the patients i.e. 66%. 

As shown in Table 1, out of total 150 cases with chronic tobacco habits, 33.3% had normal oral mucosa on visual inspection. Amongst the visually observed abnormal mucosal lesions, most common lesion was OSCC (20%) followed by Leukoplakia (18%), OSMF (14%), leukoerythroplakia (8.7%) and erythroplakia (2.6%). The most common site of involvement in OPMD and OSCC was buccal mucosa i.e. 89% and 48% respectively followed by Tongue in 6% cases of OPMD and 45% cases of OSCC. Other less frequently involved sites included palate, labial mucosa and buccoalveolar suclus comprising of 5% cases of OPMDs and 7% cases of OSCC.

After applying acetic acid in each case, change in colour of oral mucosa was noted. VIA positivity was seen in 55% cases. The percentage of VIA positivity was almost equal in leukoerythroplakia (77%), Erythroplakia (75%) and OSCC (70%) whereas it was 60% in leukoplakia and 40% in OSMF.

As shown in Table 2, cases were categorized according to the modified Bethesda Classification (Alsaraff et al., 2018) into different diagnostic groups i.e. Normal- 25.3%, ASCUS- 18.7%, LSIL- 26%, HSIL- 10.7% and OSCC-19.3%. 72.4% cases reported as OSCC on cytology were positive on VIA while ASCUS, LSIL and HSIL showed 50%, 56.4% and 87.5% positivity on VIA respectively. Out of 38 cases normal on cytology, majority i.e. 71.1% were negative on VIA. Statistical analysis of results of VIA in comparison to exfoliative cytology are shown in Table 3. There is strong association between results of VIA and exfoliative cytology in all cases (p value <0.05).

On histological examination done in 58 cases, 50% cases were malignant, 29% cases were reported as benign and 21% cases were reported as oral epithelial dysplasia. All the malignant cases were OSCC.

The findings of VIA and exfoliative cytology were compared to histopathology as gold standard. Results of VIA and exfoliative cytology showed a significant statistical correlation with histopathology (P value <0.05) as shown in Table 4. VIA in comparison to histopathology showed sensitivity and specificity of 70.73% and 64.71% respectively with accuracy of 68.97%. Sensitivity and specificity of exfoliative cytology in comparison to histopathology was 97.56% and 64.71% respectively. A higher PPV, NPV and diagnostic accuracy of 87%, 92% and 87.93% respectively were seen for exfoliative cytology.

**Table 1:** Showing Different Lesions on Visual Inspection and Results of VIA in Chronic Tobacco Users

Lesions	Number of cases (n=150)	VIA*	
		No change (n=68)	Change (n=82)
Normal	50 (33.3%)	30 (60%)	20 (40%)
Leukoplakia	27 (18%)	11 (40%)	16 (60%)
Erythroplakia	4 (2.6%)	1 (25%)	3 (75%)
Leukoerythroplakia	13 (8.7%)	3 (23%)	10 (77%)
Oral submucous fibrosis (OSMF)	21 (14%)	13 (60%)	8 (40%)
Smokeless tobacco Keratosis	4 (2.7%)	1 (25%)	3 (75%)
Palatal lesions a/w reverse smoking	1 (0.67%)	0	1 (100%)
Growth(ulcerative/fungating)	30 (20%)	09 (30%)	21 (70%)

*VIA, Visual inspection using acetic acid

**Table 2:** Cytological Categories According to Bethesda Classification and Its Correlation with Visual Inspection Using Acetic Acid

Diagnostic Groups	Cytological Criteria	N (%)	VIA	
			No change	Change
Normal	Even sized cells and nuclei	38 (25.3%)	27 (71.1%)	11 (28.9%)
Atypical squamous cells of unknown significance	Reactive changes with Mild increase in N:C ratio<10%, even chromatin distribution	28 (18.6%)	14 (50%)	14 (50%)
Squamous Intraepithelial lesion low grade	Increase in N:C ratio <50%, Nuclear membrane irregularities and anisonucleosis	39 (26.0%)	17 (43.6%)	22 (56.4%)
Squamous Intraepithelial lesion high grade	Increase in N:C ratio <50% and <75%, Nuclear membrane irregularities, Hyperchromasia and Marked anisonucleosis	16 (10.7)	02 (12.5%)	14 (87.5%)
Squamous cell carcinoma	Increase in N:C ratio >75%, Nuclear membrane irregularities, marked hyperchromasia, Marked anisonucleosis, Irregular chromatin distribution and thickened nuclear membrane, Presence of necroticc debris in the background	29 (19.3%)	08 (27.6%)	21 (72.4%)
Total		150	68 (45.3%)	82 (54.7%)

**Table 3:** Showing Statistical Analysis of VIA in Comparison to Exfoliative Cytology

		Exfoliative Cytology		Total (n=150)	
		Normal (n= 66)	Abnormal (n=84)		
VIA	No Change	41 (60.3%)	27 (39.7%)	68	P value= 0.0003
	Change	25 (30.5%)	57 (69.5%)	82	

**Table 4:** Showing Statistical Analysis of VIA in Comparison to Histopathology

	Histopathologically Negative	Histopathologically Positive	Total (n=58)	
VIA				
No Change	11 (47.8%)	12 (52.2%)	23	P value = 0.0183
Change	6 (17.1%)	29 (82.9%)	35	
Sensitivity: 70.73%, Specificity: 64.71% , PPV: 82.86%, NPV: 47.83% , Accuracy: 68.97%				
Exfoliative Cytology				
Normal	11 (91.7%)	1 (8.3%)	12	P value = 0.0001
Abnormal	6 (13%)	40 (87%)	46	
Sensitivity: 97.56%, Specificity: 64.71%, PPV: 86.96%, NPV: 91.67 %, Accuracy: 87.93%				

## Discussion

Oral cancer is one of the most common head and neck malignancies all over the world with high prevalence in South Asian countries like Bangladesh, India, Pakistan and Sri Lanka, where one-third of all the cancers reported are oral cancer (Johnson and Singh, 2011). In India a very high incidence of OPMD and OSCC is reported in Uttar Pradesh, Bihar and Jharkhand, due to higher rate of consumption of smokeless tobacco products, such as paan and gutkha in this belt (Saran and Agrawal, 1984; Mehrotra et al., 2005). In our study consumption of smokeless tobacco was more prevalent and a duration of tobacco use was more than 10 years in majority of cases. Also, it was more prevalent in males than females. The mean age of our cases was 45 years. Buccal mucosa was the most common site in OPMDs (89.1%) and OSCCs (48.3%) both, followed by lateral border of tongue. Most of the patients were from rural background. Our demographic findings were similar to previous studies. Low socioeconomic status, rural background and longer duration of tobacco habits are associated with a higher risk of OPMD and OSCC (Bagate et al., 2015; Dongre et al., 2008).

Chronic tobacco use has been associated with development of several oral lesions like leukoplakia, erythroplakia, OSMF, lichen planus and actinic keratosis. These are considered to be premalignant lesions for OSCC. Amongst the different precancerous lesions, commonest is leukoplakia with prevalence of 0.2-5.2% in India with malignant transformation rate of 0.13 to 10% (Kulkarni, 2013). In our study, most common OPMD was leukoplakia (18%) followed by OSMF (14%). Chaturvedi (2009) reported a malignant transformation rate of 17% in OSMF. Erythroplakias are the most severe of all the precancerous lesions and carry a much higher risk of malignant transformation than leukoplakia as reported by Kulkarni (2013). In our study, 2.6% of erythroplakias were recorded.

Several screening modalities have been used in the past for oral cancers, unaided VI being the most widely practiced method in India. However, in initial stages, OPMDs are often subtle and asymptomatic and can be missed on VI (Bobdey et al., 2015). To avoid missing such cases, adjunctive techniques including vital stains such as acetic acid, toluidine blue and lugols iodine can be used. In our study, out of 50 cases which were negative on VI, 40% (20/50) turned opaque on VIA. 9 out of these 20 cases showed dysplasia on cytohistological correlation. Thus, these 18% (9/50) cases, which were missed on VI were detected using VIA. 

None of the vital stains mentioned above have been validated as a definitive and cost-effective method. Although toluidine blue has higher sensitivity than rest of the vital stains, it is not preferred due to its low specificity and poor compliance because of the undue staining of oral mucosa. 

Lugols iodine is more specific than toluidine blue but it is not preferred due to its limited role in keratotic lesions of oral mucosa. Compared to the above two, acetic acid has the advantage of being cost effective, better tolerated by patients and more specific in OPMDs and OSCC as seen in this study. It acts by causing coagulation of nuclear proteins, thus a high nuclear content in premalignant and malignant lesions reacts with the acetic acid producing an acetowhite appearance (Bobdey et al., 2015).

The role of VIA in screening of cervical cancer is well established. According to a study done by Sankaranarayanan et al., (2003) the pooled sensitivity and specificity for VIA in screening of cervical cancers was found to be 82.6% and 86.5% respectively. In oral cancers, however, the utility of VIA as a screening modality has been evaluated in only few studies which have focused on malignant oral lesions (Bhalang et al., 2008; Vinuth et al., 2015). In our study we aimed to evaluate the role of VIA as a screening tool in OSCC as well as OPMDs. The biggest advantage of VIA is that, it can be implemented through primary health care workers, it does not require a well-equipped laboratory infrastructure and the results are obtained immediately following testing, thus allowing diagnosis and triaging the site for biopsy.

In our study, sensitivity, specificity, PPV and NPV of VIA was 70.73%, 64.71%, 82.86% and 47.83% respectively. For a test to be used as a good screening modality its sensitivity needs to be more than specificity as was seen in our study. The sensitivity and specificity of VIA in oral lesions has been reported as 95% and 60% by Vinuth et al., (2015) and 83% and 84% by Bhalang et al., (2008) respectively. In cervical lesions the sensitivity and specificity as reported by Singh et al., (2001) was 87% and 94%. Sankarnarayan et al., (2003) reported a similar sensitivity and specificity of 83% and 87% respectively. Though our values were lower than that given by above authors, it could be attributed to the fact that the above authors estimated the efficacy of VIA in clinically suspicious malignant lesions whereas, in our study, normal appearing mucosa, OPMDs and OSCC were also included. Also, most of our OPMD cases were of Leukoplakia and OSMF wherein the Interpretation is subjective while in inflammatory lesions, buccal mucosa becomes more permeable allowing rapid permeation of acetic acid into the cells thus causing pronounced opacity. 

To overcome the problem of low NPV with VIA, an attempt was made to combine VIA with exfoliative cytology. Cases were categorized according to the modified Bethesda Classification (Alsaraff et al, 2018) into different diagnostic groups i.e. Normal, ASCUS, LSIL, HSIL and OSCC using cytological features as already described. Sensitivity, specificity, PPV, NPV and accuracy of exfoliative cytology in the present study was 97.56%, 64.71%, 86.96%, 91.67 % and 87.9% respectively. Owing to its high NPV and accuracy, it can be used as a noninvasive diagnostic modality for OPMD and early OSCC. Exfoliative cytology when performed properly is the most accurate adjunctive technique which detects early malignant changes like dysplasia and also shows strong correlation with histopathology as shown in previous studies with a high degree of sensitivity and specificity (Remmerbach et al., 2001; Driemel et al., 2008; Mehrotra et al., 2008; Babshet et al., 2011; Jha et al., 2014; Bhandari et al., 2015; Salih et al., 2017). 

In our study 72.4% cases reported as OSCC on cytology were positive on VIA. ASCUS, LSIL and HSIL showed 50%, 56.4% and 87.5% positivity on VIA respectively. Hence, the findings of VIA correlated with exfoliative cytology. Thus, combining the two techniques can be advantageous in the way that VIA acts an effective screening tool and provides a better assessment of multiple and suitable sites from which targeted scrapes or biopsies can be taken while exfoliative cytology plays a pivotal role in detection of epithelial dysplasia for early diagnosis of malignant transformations of OPMDs. Both these tests are simple, rapid, non-aggressive and painless techniques which appear to be helpful in establishing a definite diagnosis.

Although surgical biopsy followed by histopathological examination is considered to be the gold standard in diagnosis of OPMD and OSCC, it may not be possible to obtain biopsy in every case as some of the patients may be medically unfit for an invasive procedure and a few patients with asymptomatic lesion may not give their consent for biopsy due to psychological implications. Also, repeated biopsies from multiple sites in patients with subtle changes in OPMDs are inconvenient to the patients in comparison to oral scrapes. In our study, only 40% cases agreed for biopsy. Most of these cases had an obvious growth on VI and were reported as OSCC. Any diagnostic modality is more acceptable if it is a simple, noninvasive, affordable and easily accessible method without causing much discomfort to the patients. Therefore, VIA and exfoliative cytology using toothbrush may prove to be a good alternative diagnostic tool to histology as was done in our study.

Few limitations in our study included subjective analysis of results on VIA and non-compliance in ulcerated lesions due to burning effect of acetic acid. Similarly, exfoliative cytology was not adequate in cases with scant exfoliation as in leukoplakia due to keratinisation and difficulty in obtaining scrapes in OSMF because of marked fibrosis leading to restricted mouth opening. Another limitation in our study was a small sample size and unavailability of biopsies in OPMDs. 

VIA being a cost effective method and requiring minimum technical skills, may be used for mass screening in high risk population for early detection of OPMDs and OSCC. This can also be combined with exfoliative cytology which helps in triaging the patients with increased risk of malignancy, appropriate follow up and management with improved prognosis. However, furthur studies with a larger sample size are required to establish their role in mass screening. To conclude, early detection of oral pre-malignant and early malignant lesions helps to avoid the socio-economic burden on the patient, family as well as the community especially in resource crunched health infrastructure.

## Author Contribution Statement

Author 1: Literature search, Data Acquisition, Data Analysis, Statistical Analysis, Manuscript preparation. Author 2: Concept, Design, Definition of intellectual content, Manuscript preparation, Manuscript review- Author 3: Literature search, Data Analysis, Statistical Analysis, Manuscript preparation. Author 4: Concept, Design, Definition of intellectual content, Manuscript review. Author 5: Concept, Design, Definition of intellectual content. Author 6: Data Analysis, Statistical Analysis, Manuscript preparation
